# One Medicine One Science: a framework for exploring challenges at the intersection of animals, humans, and the environment

**DOI:** 10.1111/nyas.12601

**Published:** 2014-12-04

**Authors:** Dominic A Travis, P Sriramarao, Carol Cardona, Clifford J Steer, Shaun Kennedy, Srinand Sreevatsan, Michael P Murtaugh

**Affiliations:** 1 Department of Veterinary Population Medicine, College of Veterinary Medicine, University of MinnesotaSt. Paul, Minnesota; 2 College of Veterinary Medicine, University of MinnesotaSt. Paul, Minnesota; 3 Department of Veterinary and Biomedical Sciences, College of Veterinary Medicine, University of MinnesotaSt. Paul, Minnesota; 4 Department of Medicine, University of MinnesotaMinneapolis, Minnesota; 5 The Food System Institute, LLC and Department of Veterinary Population Medicine, College of Veterinary Medicine, University of MinnesotaSt. Paul, Minnesota

**Keywords:** one health, iCOMOS, emerging infectious disease, food safety, food security, global health, One Medicine One Science

## Abstract

Characterizing the health consequences of interactions among animals, humans, and the environment in the face of climatic change, environmental disturbance, and expanding human populations is a critical global challenge in today's world. Exchange of interdisciplinary knowledge in basic and applied sciences and medicine that includes scientists, health professionals, key sponsors, and policy experts revealed that relevant case studies of monkeypox, influenza A, tuberculosis, and HIV can be used to guide strategies for anticipating and responding to new disease threats such as the Ebola and Chickungunya viruses, as well as to improve programs to control existing zoonotic diseases, including tuberculosis. The problem of safely feeding the world while preserving the environment and avoiding issues such as antibiotic resistance in animals and humans requires cooperative scientific problem solving. Food poisoning outbreaks resulting from *Salmonella* growing in vegetables have demonstrated the need for knowledge of pathogen evolution and adaptation in developing appropriate countermeasures for prevention and policy development. Similarly, pesticide use for efficient crop production must take into consideration bee population declines that threaten the availability of the two-thirds of human foods that are dependent on pollination. This report presents and weighs the objective merits of competing health priorities and identifies gaps in knowledge that threaten health security, to promote discussion of major public policy implications such that they may be decided with at least an underlying platform of facts.

## Background

With the World Health Organization (WHO) projecting that the world population will reach 9 billion by 2050, the Food and Agricultural Organization (FAO) of the United Nations (UN) estimates that the demand for food, both through population growth and economic development, will require as much as a doubling of the global food and agriculture production over the same time period.^1^ The challenges of increased food production are magnified by its potential impact on environmental sustainability and resource availability, which will require entirely new approaches to collaboration, scientific discovery, and translation of results to those who need them most. Not limited to food, the challenges of increased population include urban migration, confluence, environmental pollution, climate change, and emerging diseases, among others. The potential solutions to these challenges will require approaches that are interconnected, cross-disciplinary, and integrated.

According to the One Heath Commission, “one health” is the collaborative effort of multiple health science professions, together with their related disciplines and institutions—working locally, nationally, and globally—to attain optimal health for people, domestic animals, wildlife, plants, and our environment (https://www.onehealthcommission.org). The U.S. Centers for Disease Control and Prevention (CDC) outlines the current one health movement as a concept long recognized both nationally and globally. Since the 1800s, scientists have noted the similarity in disease processes among animals and humans, but the practice of human and animal medicine was separate until the 20th century. In recent years, through the support of key individuals and vital events, the one health concept has gained greater recognition in the public and animal health communities (http://www.cdc.gov/onehealth/people-events.html).

**Figure 1:**
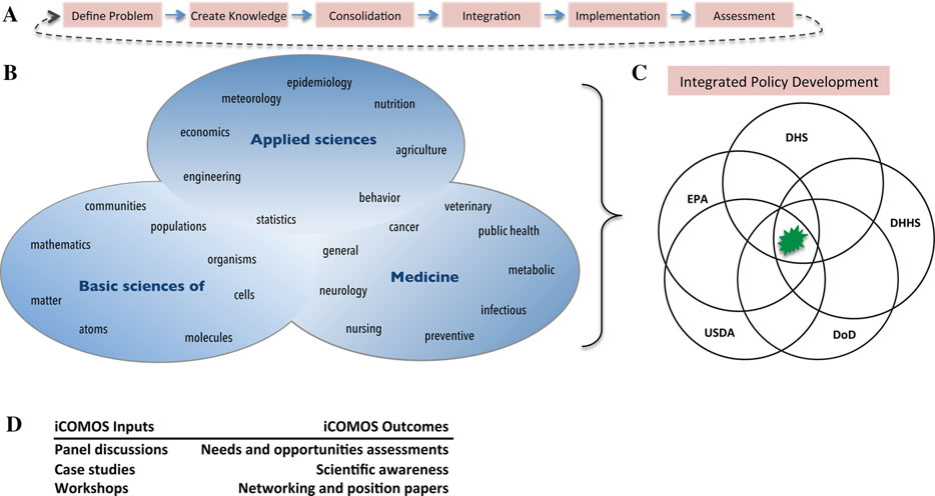
The iCOMOS: a knowledge forum melding science and medicine in a continuous process to find common ground and solutions to complex problems at the interface of animal, human, and environmental health. (A) Process flow to maintain focus on solutions at a dynamic interface. (B) Intersecting and overlapping inputs of scientific and medical knowledge that together inform durable public policy development. (C) Networking and communication nodes for integrated health policy development at the interface of animals, humans, and the environment (starburst). As an example, only U.S. government agencies are shown for simplicity. (D) Structured input and outcome elements of the iCOMOS. The overall goal is to bring together scientists, medical experts, funding sponsors, critical need partners, and policy experts locally and globally focused on preserving animal, human. and environmental health in changing times.

The University of Minnesota, a land-grant university with more than 50 multidisciplinary programs, was the original academic home of Nobel laureate Norman Borlaug, father of the so-called *Green Revolution*. It is this proud history of scientific discovery, translational research, service, and practical innovation that challenged the conveners to host a new kind of conference aimed at exploring the concept of one health as a complex and adaptive “system of systems.” As outlined in the inaugural remarks by Srirama Rao, co-chair of the organizing committee, the key objective was to highlight science that directly connects to policy and implementation strategies. Thus, the International Conference on One Medicine One Science (iCOMOS; see Supplementary Online Appendix), and its in-depth discussions of disease emergence, food security, and environmental challenges was designed to focus first and foremost on the science behind one health, but also to incorporate the linkages from both basic and applied science to the grand challenges of the modern world. These concepts are illustrated in Figure 1.

A major goal of the iCOMOS was to bring together diverse international partners from nonprofits, government, academia, and private industry to explore the science behind one health. A unifying concept, as stated by Trevor Ames and Brian Herman, was that scientific discovery and translational research play key roles in the one health approach and that there is a critical need to expand science well beyond established institutions. The conference was divided into three distinct types of sessions: plenary, panel, and workshop. Plenary sessions emphasized science and questions of disciplinary balance, with local, domestic, and international scopes. Panels emphasized the direct connection between science and policy implementation across participant sectors. Concluding workshops served as case-based studies surrounding points of particular importance to the connection between science, public policy, and private-sector implementation of real-world solutions needed to address important grand challenges in health. These included grant writing, team building and collaboration, emerging disease forecasting, and science-based policy advocacy. In addition, the conference provided networking opportunities across disciplines such as international health, agricultural sciences, environmental sciences, food security, and food safety, as well as animal welfare.

To illustrate both the global scope and complexity of these issues, a cross-cultural, multidisciplinary panel was convened during the opening evening plenary session, moderated by Maggie Koerth-Baker, a columnist for the *New York Times* magazine and science editor for BoingBoing, entitled, “Global Challenges at the Interface of Animals, Humans and the Environment: Role of Science and Discovery in the Pursuit of One Health.” In it, Peter Agre, Nobel laureate and director of the Johns Hopkins Malaria Research Institute at Johns Hopkins University, highlighted how investigating the diversity of aquaporins—water channels in cells that are present in the tissues of humans, animals, insects, and plants—led to a series of key medical discoveries in humans, including implications for malaria prevention and control.

William Bazeyo, dean of the School of Public Health at Makerere University, Kampala, Uganda, highlighted that barriers to one health still exist and that specialization, although necessary, continues to act, in part, counter to the principles of one health. In contrast, one of the successes in sub-Saharan Africa is the development of coordinating committees within countries that create an open line of communication between intergovernmental organizations, nongovernmental organizations (NGOs), ministries, and academia in order to avoid traditional political barriers to collaboration.

David Morens, senior advisor to the director of the National Institute of Allergy and Infectious Diseases (NIAID) at the National Institutes of Health (NIH), highlighted the fact that, despite more surveillance infrastructure than ever before, it is still difficult to connect scientific communities such as those working in wildlife, agriculture, and humans. More emphasis should be placed on inter- and intrasystem connectivity and targeted, purposeful surveillance research.

Lertrak Srikitjakarn, professor and dean emeritus in the Faculty of Veterinary Medicine at Chiang Mai University, focused on how the environment, both natural and artificial, affects the health of animals and people and on the need for more integrated science. This was highlighted by a case study of fasciolosis in Southeast Asia, a disease carried by liver flukes, which has a high prevalence among people in Northeast Thailand, Vietnam, and Laos. Drugs used to successfully treat liver flukes were originally developed by veterinarians for use in buffalo after finding that infection patterns were seasonal. Thus, by focusing on the treatment of animals during certain seasons, this disease may be eradicated in herds, and controlled in people. Community engagement, as well as science, was important in not only decreasing mortality in buffalo from 50% to 5% over 3 years, but also in curing human disease.

Finally, Samuel Thevasagayam, deputy director of Agriculture Development and Livestock at the Bill & Melinda Gates Foundation, discussed the role of funding agencies in trying to navigate the one health paradigm. He reported that the Gates Foundation funds many projects on diseases that affect humans, including a large focus on agriculture, particularly small shareholder farmers. Thus, the Foundation does not brand itself as “one health” per se, but rather participates actively through their actions. Examples include prevention of exotic Newcastle disease, a disease that causes up to 80% mortality of domestic poultry in smallholder farms and which greatly affects both food and economic security; as well as similar far-reaching economic and food security effects of depopulation strategies for control of avian influenza in poultry in Africa.

## Conference themes and policy-based panels

The first major theme of the conference focused on the role of science in identifying and solving health problems—attributable to infectious diseases—at the interface of humans, animals, and the environment. This was demonstrated through the presentation of a number of high-profile case studies initially focused on discovery (i.e., aquaporins and malaria, influenza, climate and land-use effect on Rift Valley fever (RVF), bovine tuberculosis as a neglected zoonotic disease), and then on the changing landscape of governmental and nongovernmental organization funding and research support. The day's discussions transitioned into solution-based examples, including vaccine strategies, AIDS treatment, and monkeypox management. David Nabarro, special representative for food security and nutrition to the UN, provided, through a prerecorded video, a high-level summary of observations and recommendations for preparing for and combating emerging diseases. The overview provided a great segue into a panel discussion on scientific partnerships for solving the global health crisis. A special evening plenary talk, “Food Animal Domestication and Human Advancement,” was presented by Sonny Ramaswamy, director of the National Institute of Food and Agriculture (NIFA) of the U.S. Department of Agriculture (USDA), who highlighted the important theme of human–animal connectedness in society, and previewed discussions regarding food security for the following day.

The second major theme of the iCOMOS focused on the role of science and the one health approach in providing sustainable food production and safety. Again, scientific presentations accompanied by panel discussions served to connect scientific issues to the policy-based relevance and implementation of the science. The importance of food production as a “grand challenge” was introduced by Allen Levine, vice provost, faculty and academic affairs, University of Minnesota (UMN), and expanded by Mehmood Khan, executive vice president and chief scientific officer of PepsiCo. The plenary presentation was followed by a panel discussion entitled, “Meeting the Challenges of Feeding the World,” which again highlighted the complexity of this issue from the perspectives of private food companies. Scientific presentations included the interplay between modern food-production methods and the environment, the current global challenge of honey bee health, and a one health perspective on antibiotic resistance.

The grand challenges of food safety were introduced by Will Hueston, professor of veterinary preventive medicine and endowed chair of the Global Institute for Food Systems Leadership in the Center for Animal Health and Food Safety at the UMN. Catherine Woteki, USDA chief scientist and undersecretary for research, education, and economics, laid out the USDA approach to addressing these challenges in the United States and as a global citizen; and Stanley Maloy, dean of the College of Sciences at San Diego State University, discussed the evolution of microbes, primarily *Salmonella*, from environmental adaptor to food safety hazard. Michael Apley and Henrik Wegener laid out the challenge of antibiotic resistance from divergent perspectives. Jennifer Kuzma discussed attitudes toward foods with genetically modified and nanotechnology ingredients and expectations of safety, a concept that cannot be based solely on science, but includes the values of the community setting safety standards. The conference program was summarized by Michael Murtaugh, co-chair of the organizing committee, with an eye toward the future role of One Medicine One Science in addressing complex health challenges.

### Role of science in solving emerging infectious disease threats at the interface of humans, animals, and the environment

Zoonotic diseases represent important health issues for humans and animals and exemplify the need to connect science to policy and ecosystems to human society. Basic experimental science aimed at identifying and characterizing a disease agent may lead to the development of hypotheses for control strategies, while applied science such as human and animal health surveillance contributes to the evaluation of control policies. Each provides much-needed data for risk assessment–modeling efforts on which policy is often based. Applied research connects directly to communities and includes molecular epidemiology techniques that link micro- to macroperspectives on the front lines of disease emergence. The result is a better understanding of transmission risks of emerging and neglected diseases such as RVF, *Mycobacterium bovis*, and Ebola virus. In this forum, each speaker reinforced the fact that (1) science must focus on the real-world impacts of disease on society and those aspects that lead to disease prevention and control; and (2) that studies must be more frequently linked to the causal interface between animals and the environments in which they live.

Case studies from East Africa—*Mycobacterium tuberculosis* in Tanzania and RVF in Kenya—reinforced these points while providing real-world examples. Rudovick Kazwala, a professor in the Department of Veterinary Medicine and Public Health at Sokoine University of Agriculture, Tanzania, and his associates have extensively explored the importance of *M. bovis* to both animal and human health. In his presentation, Kazwala focused on efforts to untangle the ecology of tuberculosis in pastoralist societies.^2^,^3^ This involves a multipronged approach aimed at characterizing the contribution of *M. bovis* in the overall ecology of tuberculosis, understanding the social and political aspects of water availability and water usage rights, assessing the impact of wildlife maintenance hosts and reservoirs, the effects of land-use policies (both conservation and grazing rights) on wildlife–domestic cattle interactions, and the social and economic aspects of cattle ownership and economics in pastoralist societies.

The 2006–2007 RVF outbreak in Kenya provides an exemplary case for the development of a one health approach connecting science and policy at the community and national levels. As M. Kariuki Njenga, head of the Integrated Human–Animal Health (One Health) Program, at the CDC–Kenya, detailed, in mid-December 2006, the Ministry of Health in Kenya received reports of fatal cases of a febrile hemorrhagic illness of unknown etiology among people living in the Garissa district in its northeastern province, after unusually heavy rains and flooding in the area. The outbreak included over 700 suspected human cases (392 confirmed) and 90 deaths; there were up to 180,000 infected mildly ill or asymptomatic people within highly affected areas.^4^ Compared with previous RVF outbreaks in Kenya, the 2006–2007 outbreak was the most extensive in cattle, sheep, goats, and camels, affecting thousands of animals in 29 of 69 administrative districts across six of the eight provinces. This contrasted with the distribution of human cases, where over 85% were located in four districts. Analysis of livestock and human data suggested that livestock infections occur before virus detection in humans, making human–animal integrated surveillance a possible prevention strategy.^5^

Endemic and epidemic cycles of RVF are closely associated with heavy rainfall and the presence of *Aedes* and *Culex* mosquitoes, the most common vectors. Epidemics often occur in conjunction with El Niño/southern oscillation climatic phenomena. Environmental and climate models using satellite data have been created in an attempt to predict areas where outbreaks of RVF in humans and animals may be expected. In 2005, these models correctly predicted RVF outbreak conditions in the Horn of Africa, Sudan, and Southern Africa for the period 2006–2008.^6^ Interestingly, risk factors for infection and illness in this outbreak did not include mosquito exposure, but identified the consumption and handling of products from sick animals and being a herdsperson; touching an aborted animal fetus; and consuming or handling products from sick animals. Although (mostly male) abattoir workers, veterinary personnel, herdspersons, and farmers are often identified as high risk, Anyangu *et al*. found a high proportion of acute RVF infections in housewives, thought to be connected to their handling of sick animals and/or products during food preparation.^7^

Like *M. bovis*, cultural practices may play a larger than normal role in the RVF story, since the nomadic lifestyle and traditional beliefs of communities living where most outbreaks occur may directly influence the ecology of this disease as well as human exposure risk. With the onset of seasonal rains, many pastoralists move their herds to areas with new grass growth and standing water. As the rains continue, herds are moved away from the increasing number of mosquitoes around the flooded areas, potentially introducing infection into new areas. The fact that pastoralists accompany their animals into mosquito rich areas, and that communities slaughter and consume the meat of ill or recently dead animals to salvage the protein, means that pastoralists are at direct risk for contracting this disease through numerous routes.^5^

The 2000 RVF outbreak in Saudi Arabia led to trade bans of live animals from Ethiopia, Somalia, and Kenya, with devastating economic impact. In this region, livestock and livestock trade may represent over 90% of a pastoralist's income. The 2007 RVF outbreak in Kenya also had wide-ranging economic impacts, due mostly to loss of production, employment, and casual labor, as well as reduction in operating capital among slaughterhouses and butchers. Rich *et al*. estimated that RVF resulted in losses of over U.S. $32 million and is a lesson in the need to fully evaluate the economic impact of this kind of outbreak by assessing the damage to the multiplicity of stakeholders that are affected.^8^ The impact of disease on the food-supply chain and costs to other parts of the larger economy often dwarf the impacts of the disease at the farm level, but public policy tends to concentrate primarily on losses accruing to producers.

In 2011, the RVF experience, combined with the global emphasis in preventing H5N1 influenza, stimulated Kenyan experts and politicians to establish the National Zoonotic Disease Unit (ZDU) to provide leadership, expertise, and service in laboratory and epidemiological sciences, bioterrorism preparedness, applied research, surveillance, outbreak response, and policy formation. Njenga explained that the mission of the ZDU is to provide an effective, efficient, multidisciplinary and multisectoral surveillance and response system that reduces the burden, risk, and spread of zoonotic diseases in Kenya. This network is further supported by One Health Central and East Africa, a network of schools of public health and veterinary medicine dedicated to the training of a future work force capable of implementing the one health paradigm.

Infectious diseases are by their nature dynamic processes that come and go throughout the landscape, both on imperceptible and grand scales. Regardless, emerging diseases or spillover events have increased their occurrence, or at least we have an increased ability to recognize them.^9^ If they are indeed increasing, this is at least in part due to anthropogenic perturbations of the system such as eradication of pathogens (e.g., smallpox and *Salmonella pullorum*), land-use choices, unsustainable protein sourcing from wild animals, and over-use of natural resources. Monkeypox provides a unique case study of “slow-motion” emergence (over decades), which is ideal for developing methods that can be applied to other prepandemic pathogens.^10^ In this case, models were useful in making conclusions about epidemiology with limited data. Graphs allowed stakeholders and policy makers to visualize the probability of spillover and outbreak size from various starting conditions.^11^ There is a need for more study of the evolution of pathogens to understand evolutionary processes, including the spillover events across species barriers, and to develop the scientific tools needed for the next century of emerging disease surveillance.^15^

It is clear that additional tools are needed to handle the complexity of emerging diseases or spillover events. Richard Webby pointed out in his lessons learned from influenza that, although surveillance is widely recognized as important, exactly when and where to watch for emergence and what to do with the vast amounts of information remain a challenge in this area. For example, influenza viruses of interest and concern might be identified from surveillance activities in Southern China, but predicting the risk of human disease from an isolate is beyond our capacity. The movement of viruses across species barriers remains almost entirely unpredictable, underscoring the importance of addressing these issues for diseases that are emerging at an alarming rate. In fact, dynamic models for macroscale integration are within reach, but there is a real need to develop methods to integrate microscale knowledge. The genomics explosion in sequencing and bioinformatics has generated enormous amounts of data, but there are limited resources to integrate genetic changes in a pathogen, for example, with multiple biological, environmental, and population variables.

While scientific tools are needed to understand our world, political strategies are needed to manage the increasing amount of information being derived and released to the public without context in ever-shorter news cycles. Political will is needed to prioritize and manage disease preparedness and prevention efforts in the face of a reactionary approach to these issues. When risk is perceived as low, political will and investment are low; and when risk is perceived as high, enthusiasm for investment is high. David Nabarro identified that a more defined level of consistency is necessary to address risks at all levels. The cost of outbreaks in response, loss of productivity, and injury to humans, animals, and the environment is far more costly than prevention and preparedness, but must be “sold” without exaggerating risks. A systematic approach to scientific discovery will require consistent political support in light of the inconsistency of risk. The U.S. President's Emergency Plan for AIDS Relief (PEPFAR) is an example of continuous political will resulting in progress in disease control in many areas of the globe.^12^

HIV continues to be a major public health issue in many African countries, and limited financial resources remain a challenge. PEPFAR and other initiatives have funded efforts in 15 countries to provide antiviral treatment to infected individuals, improved community medical-service infrastructure, and training of medical professionals. This provides an excellent demonstration of the role of science in providing applied solutions for public health. Treatment efforts include strong monitoring programs aimed at assessing efficacy and emergence of drug resistance. Phyllis Kanki, Department of Immunology and Infectious Diseases at the Harvard School of Public Health, noted that one of the notable areas of success in this arena has been in reduction of mother–child disease transmission. Limited drug choices have led to a growing interest in characterizing drug resistance among HIV subtypes. To address the problem of drug resistance, a new polymerase chain reaction (PCR) technique was developed that can detect subtype-specific mutations associated with drug resistance. More importantly, it is being adapted to an enzyme-linked immunosorbent assay (ELISA) test that can be used in hospitals to help make appropriate real-time treatment decisions for individuals. PEPFAR and other public–private partnerships exemplify collaborative efforts toward the common goal of improving public health. The key focus is on community involvement and building local resources that can be applied not just to HIV/AIDS, but to other diseases as well.^12^ A medical education partnership initiative with African medical schools is designed to modify the medical curriculum and incorporate concepts of one health and team medicine, including community involvement and cooperation between schools of medicine, pharmacy, nursing, and veterinary medicine. HIV/AIDS and the sustained PEPFAR initiative are examples to demonstrate that the combination of research with capacity building and training programs provides many future opportunities for improving health in a sustainable way.

#### Scientific partnerships for solving the global health crisis panel

A panel moderated by Brian Herman, vice president for research at the University of Minnesota, rounded out the discussion of the role of science in solving emerging infectious disease threats at the interface of humans, animals, and the environment. Panelists included Andrew Clements, deputy director of the Pandemic Influenza and Other Emerging Threats Program at the U.S. Agency for International Development; Carole Heilman, director of the Division of Microbiology and Infectious Diseases at the NIAID; Sonny Ramaswamy, director of the NIFA under the USDA; and Samuel Thevasagayam, deputy director of Agricultural Development and Livestock at the Bill & Melinda Gates Foundation.

During the panel discussion, it was recognized that an expanding global population, the increasing threat of zoonotic spread of infectious diseases, and environmental resource depletion are leading causes of the global health crisis. Furthermore, no one person, no one alliance, and no one nation is equipped to solve the problem. It is only by forming strong scientific partnerships that we will be prepared to withstand the challenge. Scientific partnerships not only improve our ability to understand disease, such as pandemic influenza and antimicrobial resistance, but also result in shared resources that can be translated to meet the needs of end users. For example, the recent partnering of veterinarians to better understand the ecology of pandemic influenza and to improve intervention systems by surveying livestock is allowing us to potentially detect pandemics before they occur. The next step is to improve the platform that has been developed and transform risk mitigation into prevention by generating a more focused picture of disease spillover from animals to humans. In another example, the timely collaboration between the NIH Division of Microbiology and Infectious Diseases, the Bill & Melinda Gates Foundation, other international supporting organizations such as the Medical Research Council in England, and private sector partners enabled the successful development and distribution of a pneumococcal conjugated vaccine that saved the lives of many children in Gambia. The collaborative effort allowed each participating organization to commit their individual strengths and constraints. Only through partnership could they overcome logistical, monetary, and scientific obstacles. This example shows that interdisciplinary approaches that are also multiorganizational can be key factors in solving the global health crisis. Multidisciplinary and multi-institutional scientific efforts that translate complex data and deliver it to the end user is a prerequisite for success.

The global health crisis is in part driven by the need to feed and provide shelter, clothing, and fuel to the world without diminishing land and water resources, changing incomes and diets, or increasing urbanization. The conundrum of meeting the challenges of food security while at the same time improving health outcomes lies at the heart of this “wicked problem.” There is a need for transformative studies that can lead to knowledge and be translated into innovation, but the process of obtaining funding can be a significant barrier to transformative research. Discovery research comes with high risk and high reward. Since many funding agencies use strict guidelines as a metric, the peer-review process can sometimes become an obstacle to innovative scientific discoveries. There is a need for scientists who favor high-risk, high-reward research to become peer reviewers in order to help shift the conservative culture of the funding process.

The panel agreed that, while it is true that scientific partnerships are needed to address the global health crisis, barriers to collaboration are not trivial at the funding level. For instance, in the most recent RVF outbreak, there were plenty of donors and funds to conduct the work, but the requirements from each donor were different enough to hinder collaborative scientific and medical efforts. This was a clear example of the importance of donor collaboration in order to match support with the needs on the ground. Detailed discussions and agreements on issues that bring together groups with differing missions and priorities is ultimately key to successful implementation. Funding agencies are beginning to recognize the need for and value of scientific partnership and are starting to address the funding barriers that exist. For instance, the Interagency Policy Committee has mandated new strategic plans that require collaboration between agencies to solve antimicrobial resistance challenges.

Finally, the lack of young people entering the biomedical sciences is clearly a concern across disciplines and across funding agencies. This may be due, in part, to the lack of resources that are available to help fund the next-generation workforce. A shortage in the biomedical workforce could have potentially devastating consequences in the future if there is no one to take the place of productive, albeit aging, investigators. Added to the challenge is the fact that the current educational system may be developing a cadre of people who are similar in their thinking, which may not be the best path to innovative science. Action must be taken across disciplines to ensure that young people today are incentivized to enter the biomedical workforce so that tomorrow's experts in human medical science, veterinary science, and environmental science are able to develop effective and innovative approaches to the grand challenge of health.

#### Food-animal domestication and human advancement

In his plenary presentation, Sonny Ramaswamy (director of the NIFA) connected the two themes of the conference. In his address, he spoke of four major topics, the first of which was a new economy. He spoke of the transition toward an information-based economy and the impacts of resource scarcity, sustainability demands, and globalization and how these frame our lives and the world we live in. The second topic he covered was so-called wicked problems, and he drew an illustration from the works of Nobel laureate Richard Smalley, who in his world travels found that challenges such as poverty, environment, education, water, climate change, and food were common themes from his audience regardless of geographic location. His third topic was the 21st-century food-system challenges, one of which was the disinterest and lack of incentives for the next generation of potential farmers. Approaches to address these challenges included ensuring competitiveness of agriculture, mitigating the ecological footprint in the face of climate change and resource limitations, tapping into the bioeconomy of what plants have to offer, and combating health challenges of chronic diseases that are a result of our diets and behaviors. On the other end of the spectrum, we have the challenges of food security and ensuring that there is sufficient safe and nutritious food available to drive positive health outcomes. The fourth topic covered was the One Medicine One Science approach to addressing these wicked problems. When engaged in research, it is important to consider other disciplines or approaches to a problem of concern. He provided the example of infectious disease researchers who looked at the impact of nutrition on health outcomes in HIV^+^ people and found that improved nutritional status correlated with improved health outcomes among people living with HIV. Additionally, he proposed tools that could help improve the efficiency and impact of the one health approach, such as the development of communities of practice using the extension model, drawing from the integrated pest-management approach that is based on prevention, monitoring, surveillance, and suppression, and borrowing from the Leroy Hood paradigm that seeks to develop personalized medicine based on the predictive ability of large dataset modeling.^13^ He concluded with a discussion on the challenges of training and working with current and future generations in which social media and digital circuitry promote linear thinking and a lack of focus. Our future leaders will require an approach that addresses these wicked problems in a new economy.

### Alignment of safe food, sustainable production, and consumer attitudes

The quest for food in the quantity and with the qualities we desire drives a major political question of modern existence. Conflicts over limited environmental resources, technology acceptance, antibiotic use, food security, and the right to free choices among all possibilities are central to the debate. The iCOMOS examined these issues through a series of case studies that pointed out the complexity of the grand challenges facing modern food and agriculture. The increased prevalence of antibiotic resistance, for example, points out that there are no simple answers to the complex movement of microbes and genes between humans, animals, and the environment. Demand for food from an increasingly wealthy and ever-expanding human population calls for improved productivity that, at the same time, is limited by some consumers’ fear surrounding genetically modified foods (GMOs) and the like. Strategies that have singularly pursued the demands of human needs without consideration for animals and the environment have had unintended negative consequences. Cultivating monocrops and increasing farm size, for example, have improved the availability of inexpensive food but have stressed bee colonies to collapse. Every current strategy for fulfilling the future food needs of the world has undesirable consequences, reinforcing the need to find fundamentally new and different approaches that better meet food security needs with maintenance of animal and environmental health.

There are no simple solutions. Antibiotics once seemed to be a universal, targeted solution for human and animal health. Henrik Wegener, provost and executive vice president of the Technical University of Denmark, noted that, faced with widespread resistance, there seem today to be two schools of thought to either (1) continue with unrestricted use with the expectation that new antibiotics will be discovered; or (2) restrict use to preserve what we have. The bulk of the large volumes of antibiotics used globally in food animals are relatively old and the contribution of their use in food animals to resistance in human infectious disease is complex and difficult to quantify, as also pointed out by Michael Apley, a professor at Kansas State University. Still, the global supply of food is so interconnected that the opportunity exists to move both antibiotic resistance genes and microbes globally through unique concentration points in the food and agriculture system. For example, only a few poultry flocks provide all the birds to industrial farms globally. This type of consolidation could be an efficient way to distribute resistant organisms worldwide through the movement of microbes and genes. Still, the food-production system today does a good job of protecting most people from resistant microbes through the consumption of food, although the role of antibiotics in agriculture in increasing the prevalence of antibiotic-resistant organisms may be significant. Apley remarked that the large denominators associated with the use of antibiotics in food animals puts their use into the correct context. Hence, keeping the number of animals treated low is an important part of the strategy that can be linked to good producer–veterinarian interactions and can effectively be used to address antibiotic resistance. Because food is connected globally, collaboration of all sectors, including consumers, to address these questions is essential. Balancing the uses of antibiotics will be integral for future food production, as will be understanding and reducing the risks that antibiotic resistance in pathogens pose to humans. To some, that solution will be dependent on the relationship between the producer and the veterinarian. For all, the increasing global demand for animal protein is an undeniable reality. The amount of antibiotics used to yield a kilogram of meat varies widely across countries and across production systems. A first step might be to understand whether these differences are justified and to understand that there are no solutions to this paradox, only common ground that considers humans, animals, and the environment together.

Catherine Woteki, undersecretary and chief scientist of research, education, and economics at the USDA, and Stanley Maloy, dean of San Diego State University, observed that the flow of disease between animals and humans is bidirectional. Humans clearly have the capacity to both increase and decrease food safety challenges and, as the food requirements of the world increase, food production systems must promote technological growth to increase agricultural efficiency and to maintain a safe food supply. But the lessons from the past demonstrate that growth requires the careful consideration of the needs of humans, animals, and the environment. Eradication of *S. pullorum* and *S. gallinarum* from commercial poultry dramatically improved bird health. *S. pullorum* and *gallinarum* killed baby chicks by the millions worldwide, and their elimination has greatly improved the efficiency of poultry production and animal welfare. Stanley Maloy showed that, once removed, a microbial niche was created for another *Salmonella* serovar, *S. enterica enteritidis*, which was not previously bird adapted or safe to humans. It was discovered through public health surveillance and, as a result, an equivalent animal monitoring system was developed. This experience demonstrates that a primary mechanism to improve the safety of food products is increased surveillance of food-production systems that involves multiple interests such as public health officials, human and veterinary doctors, wildlife experts, and food producers, which can greatly improve responses to failure by eliminating redundancies and improving early detection of the event. This is inherently a one health approach, as it requires integration across animal, environmental, and human health to ensure the safety of the food supply.

Food safety is not only vulnerable to existing threats but also to those that are emerging. Science becomes a mechanism to either identify or understand the threats, as illustrated by the use of basic science to demonstrate unexpected pathways for food-borne *Salmonella* illness outbreaks. Stanley Maloy's fascinating story showed that early tracing back of food-borne illness outbreaks associated with *Salmonella* almost exclusively linked them to foods of animal origin owing to cross-contamination during slaughter and processing, mishandling of product during preparation, or chicken eggs. In the 1970s, however, food-borne *Salmonella* illness outbreaks were associated with vegetable produce for the first time.^14^ Fecal contamination through the use of manure as a fertilizer was a proposed mechanism. Based on this assumption, mitigation strategies focused on thorough washing or dipping in sanitizers, but they only had limited success. *Salmonella* outbreaks began occurring with links to fruit and other produce products with no evidence of cross-contamination from animal waste. The limited success of intervention strategies and continued outbreaks raised a fundamental question of how fruits and vegetables could serve as vehicles for the transmission of *Salmonella* to humans.

While it was known that organisms could become entrained in plant tissue through contaminated water entering cracks in the plant tissue or natural entry points such as stems, this phenomenon still did not explain all of the outbreaks. By studying plants that were exposed to or contaminated with *Salmonella*, researchers discovered that the bacterium was not simply absorbed by the plant via contaminated water, but demonstrated that *Salmonella* actually grows interstitially in the plant tissues if the plants are in an active growth phase. Plants not undergoing photosynthesis for growth do not provide the same growth niche. Given the rate of *Salmonella* growth in plants and the presence of certain genes, this capability was most likely acquired through horizontal gene acquisition from plant pathogens. The regions in the U.S. southwest and northern Mexico where the gene transfer most likely first occurred often subject plants to reduced water stress, thus most likely increasing the selection pressures on *Salmonella*. Bacteria that acquired plant pathogen genes are thought to have facilitated *Salmonella* colonization and the ability to survive water stress. This finding explains the unexpected association of *Salmonella* with fruit and produce items where even low-level exposure of food plants to *Salmonella* can be a significant food-safety risk, as anything short of cooking will not inactivate the pathogen once it has grown in the plant. This significantly changes food-safety risk-mitigation strategies. That bacterial virulence factor genes can be found in unexpected organisms or in environments physically distinct or distant from disease outbreaks suggests that bacteria pick up genetic material by horizontal gene transfer more readily than had been thought, and that the mechanisms for geographical spread are potentially very complex.

While agricultural researchers and producers are actively engaged in food-safety risk management, consumers are less well informed, particularly in relation to emerging technologies. Jennifer Kuzma, Goodnight-NC GSK Foundation Distinguished Professor in the Social Sciences and co-director of the Genetic Engineering and Society Center at North Carolina State University, discussed U.S. and European policies toward GMOs and nanotechnology. She noted that consumers are increasingly suspicious of a lack of transparency and communication about the possible risks of the food they eat, and that these concerns are heightened when new technologies like GMOs are involved. She presented her recent research, in which GM food labeling is given high priority and different consumer types emerge based on technological comfort and health benefits as perception factors. So, despite calls for technology growth from Catherine Woteki, the impacts on the consumer market are unclear. Consumer research suggests that there is a stratification of attitudes ranging from concern with price differences over safety, to consumers who would never purchase these products owing to perceived safety concerns. While these groupings suggest that it is impossible to please every consumer, they also demonstrate that a subset of consumers would like all products to be labeled with access to their information through a variety of transparent sources. In the absence of accessible and clear information, many, possibly most, consumers are suspicious, and this is increased with inconsistency among scientific opinions and public policies.

While the U.S. food and agriculture system is predominantly managed by the private sector and is subject to market pressures, it is also regulated by government agencies and intergovernmental organizations. Today, the private sector feeds 98% of the world. However, as the global population will increase from 7 to 9 billion before 2050, both Mehmood Khan and Jonathan Foley, past director of the Institute on the Environment at the University of Minnesota, who represent different ends of the private and public sectors, agreed that there is a critical need for increased food and water supplies now and in the future. With millions going hungry daily and dwindling water sources, food insecurity, and water conflicts represent an ever-growing problem. It is estimated that 2–3 billion people will go hungry in 2050. This challenge will not be met through increases in crop yields alone, and the potential for significant expansion of productive farmland is limited. Given the scarcity of available cropland for expansion, agricultural expansion is actually impinging upon, and threatening, tropical habitats in some countries. If the focus is human needs alone, food production will increase, to the detriment of the environment. Solving this critical issue will require simultaneous advances in optimizing human diet, protecting land use, and improving agricultural practices. A solution to this problem will undoubtedly require global collaboration.

iCOMOS elucidated the unintended consequences that arise from an imbalanced pursuit of one goal without the holistic consideration of contributing resources. Focusing on only future food production would likely lead to conflicts over global water use, increased deforestation, and suboptimal soil conditions. Further from the headlines, Marla Spivak, MacArthur fellow and professor at the University of Minnesota, illuminated the struggle of honey bees to overcome the stressors imposed by modern agriculture. Bees are integral to fruit and nut production, and pollinate as much as two-thirds of human foods globally. Over the past 60 years, honey bee populations have been on an unsustainable decline. Crop monocultures provide poor bee diets, and shifts in agricultural and land-use practices limit the sustainability of bee populations worldwide. Human food production demands are inextricably linked with honey bee health, but are often in conflict with modern agricultural use of insecticides and pesticide additives that are inadvertently spread to hives.

Independent of food production challenges, food wastage in developing nations can be up to 70%, owing to postharvest losses and poor infrastructure, while about 40% of food supplies are wasted in developed nations, mostly at retail and in the home. There is an obvious opportunity to develop and implement better infrastructure systems to redistribute food to those people who are lacking and ensure more efficient use of food resources. Most importantly, Jonathan Foley suggested that more food does not necessarily need to be produced at the global level; current food supplies may be able to feed the world, if waste is reduced and dietary demands are modified to minimize the need for resource-intensive foods. Only cooperative efforts by industry, academia, government, and consumers will provide effective solutions to reduce food and water losses. Mehmood Khan, Jonathan Foley, and Marla Spivak together proposed global strategies that limit postharvest waste; find new ways to more efficiently produce food that balance human, animal, and environment needs; and rethink human diets as the most reasonable approaches to rebalance sustainability.

#### Alignment of safe food, sustainable production, and consumer attitudes panel

A panel discussion provided insights into the translation from research and policy to implementation by practitioners. It was moderated by Shaun Kennedy, director of the Food System Research Institute, LLC and adjunct associate professor of food systems, University of Minnesota, and Allen Levine. Panelists included Sarah Brew, a partner and food litigation and regulatory practice lead at Faegre Baker Daniels; Erin Fitzgerald, senior vice president of sustainability at the Innovation Center for U.S. Dairy; Mehmood Khan; and Mike Robach, vice president of Corporate Food Safety, Quality & Regulatory Affairs at Cargill, Inc. Through moderator and audience questions, they explored the actions that industry is implementing or considering, including policy changes that would make access, affordability, and choice more easily achievable within the global food system.

It is often overlooked when considering the role of industry in mitigating human, animal, and environmental risks that many of the strategies to reduce risks are also good for business. Mehmood Khan explained that PepsiCo's investments in reducing the sodium, sugar, and oil use in their products makes the products healthier and also yields significant cost advantages. In responding to the role of the dairy industry in greenhouse gas emissions, Erin Fitzgerald outlined efforts to introduce and expand conversion of manure and waste into biofuels that resulted in both reduced disposal demand and lowered fuel costs. By providing organically produced product options, Cargill is meeting the demands of a subset of consumers while also expanding their options in the marketplace.

There were similar examples of dual benefit opportunities in nutrition. PepsiCo's Project Asha in India (Mehmood Khan) and the U.S. dairy industry's Fuel Up to Play 60 program (http://www.fueluptoplay60.com/) are both centered on improving nutrition and reducing hunger through healthful products and education for children and adolescents. These efforts also resulted in increased product and brand awareness among future consumers, even though that was not the primary goal. Nutrition is an area, however, where the educated participation of all stakeholders must be enhanced. Mike Robach and Mehmood Khan pointed out that the well-intended initiatives of food manufacturers to change sodium, fat, or sugar content have often faced initially negative consumer responses, only to be reversed through education. All panelists agreed that the attempts of policy makers to be current with the state of the science can lead to regulations and dietary guidelines in constant flux, confusing consumers and frustrating industry. In some cases, as explained by Mehmood Khan, good options for health like palm oil represent an environmental threat owing to potential conversion of rainforests to plantations.

Food safety represents a different set of challenges driven by history, regulatory regimes, and litigation. Some food-safety regulations are not based on science, while others are influenced more by risk of litigation than by public health benefit. These practices often may exacerbate the problem of food waste. The difficult balance of technology, safety, and health is also apparent in such areas as GMOs. Technologies that can improve sustainability and human health while reducing chemical use also pose a range of real and consumer-perceived risks to humans, plants, and animals. The panel closed with agreement that there are no perfect answers, and it is only through public–private–academic partnerships that acceptable solutions will be found to any of the challenges facing the food system.

## iCOMOS workshops: translating science and medicine into policy

Four themes for implementing One Medicine One Science were identified and used to focus workshops that came at the end of the iCOMOS meeting. “One Health Partnerships: the Reality on the Ground,” was jointly convened by the UMN School of Public Health and the College of Veterinary Medicine; “Is it Possible to Predict the Next Outbreak Threat?” was convened by Srinand Sreevatsan from the UMN College of Veterinary Medicine with collaborators from the UMN School of Medicine and the School of Public Health; the “Vision for Safe Food in a Food System” workshop was jointly convened by the iCOMOS, the Minnesota Governor's Food Safety and Defense Task Force, and the Minnesota Department of Agriculture, with participants from industry, academia, and government; and “Grantsmanship in an Changing Environment” was a joint venture between the iCOMOS and the NIH.

### One health partnerships: the reality on the ground

“Synergy, understanding, and collaboration” emerged as the three most common descriptors of the benefits of one health partnerships. Participants explored the reality of one health partnerships in a day-long workshop, listening to case studies of successful one health partnerships and sharing the experience of working across disciplines and sectors to address complex problems. By the end of the day, participants agreed that they were more comfortable working with other disciplines and sectors in applying the one health approach to complex challenges as a result of the workshop. In addition, almost all felt they had broadened their personal networks and gained better insights into the key requirements for successful one health partnerships.

Five case studies of successful one health partnerships set the stage for the workshop. The first scenario highlighted the importance of organizational design to foster a collaborative work culture, provide opportunities for interactions among agencies and disciplines, and create teams of personnel with a variety of backgrounds and experiences. The second scenario described a way to build critical trust among partners through collaborate research grants in order to later allow open discussion of biosecurity between scientists from numerous countries. An example of the H5N1 initial outbreak in Thailand showed the importance of engaging communities through attention to concerns and viewpoints and inviting a variety of disciplines to come together when approaching the communities. The importance of strategic planning to stimulate collaboration through co-location was illustrated by recounting the events leading up to the construction of a building to house the State of Minnesota Department of Agriculture and Department of Health in shared space. Finally, a panelist described his experience throughout the initial West Nile Virus outbreak in the United States, where the disconnect between human and animal disease surveillance was overcome by dogged determination and the sharing of scientific information, eventually leading to a synergistic surveillance.

Assigning the participants to the task of providing recommendations on animal exhibits to the State Fair Board provided a hands-on exercise in one health approaches. Key resource experts shared past experiences with disease spread from animals to humans, from humans to animals, from animals to the environment and then to humans, and, finally, avoidable occupational injuries. Small groups toured the Minnesota State Fairgrounds, interviewing a fair official and visiting animal barns and the “Miracle of Birth” center. Subsequent discussion included the enumeration of the multiple perspectives needed to understand the setting, brainstorming approaches for increasing hand-washing among children, and a reality check that not all ideas that sound good on paper are implementable. Experts from the UMN and the Minnesota Department of Health shared national efforts to develop compendia of best practices, proactive training of fair personnel, and the need to weigh competing risks when working with animal exhibits, given the recognized psychosocial benefits of these human–animal interactions balanced with the risks of disease transmission or injury. The participants concluded the workshop by sharing their characterization of the benefits of one health partnerships. The importance of open, trusting relationships was highlighted.

### Is it possible to predict the next outbreak threat?

This workshop was designed to challenge existing paradigms relative to tools, technologies, and systems used in infectious disease emergence and/or reemergence investigations based on an analytical review of key concepts. Applications of genomics and spatial epidemiology in active surveillance and as predictors of new disease emergence were posed to the audience. The ability to perform gain-in-function studies derived from existing data and to simulate probability of disease emergence was discussed.

#### Microbial population structure and functioning

The ability to distinguish a symbiont or saprophyte from a parasite was identified as one potential advancement. The human body contains three times as many bacterial cells as human cells. For 10 years or more, we have tried to understand the complexity of the microbiome. This is further complicated by variation within and between host individuals. The microbiome is like a whole organ in the body, making it very difficult to predict how microbial population shifts affect the collective physiological contribution. In addition, the effect of changing terrain or ecosystem perturbation in terms of stressors and available nutrients that may lead to changes in the lifestyle of a host individual in a population was identified as a black box.

#### Mathematical models as predictors of outbreaks

It was proposed that *de novo* mathematical models could help predict the next outbreak. The caveat was that they must be integrated with good surveillance systems that are currently based on a priori knowledge of pathogens and disease patterns. An argument that natural patterns and shared mechanism data could be used in outbreak prediction was made. Predictions can be both positive and negative, but the overall outcome is to cause positive change. Models can help guide epidemiologists and ecologists on data collection.

#### Existing diagnostic paradigm

Macro- and microscale evaluations are both considered in disease investigations. Diagnostic testing needs to be carried out in a stepwise manner, and standardization of care is very critical. When an individual comes into a clinic, the clinician needs to figure out what the problem is on a macroscale, but for this, the clinician needs to look to the population to carry out effective disease surveillance. For example, in some parts of Africa, when an ill patient presents, the physician looks for the common diseases in the area to determine the tests to be run and the proper range of specificity or sensitivity. Cost is a major consideration as well, since expensive tests are unrealistic. Another important question is whether to look for unknown or known pathogens. The fragmented medical system in the United States can make it difficult to follow up in cases with multiple levels of health personnel, whereas lack of diagnostics in underresourced countries contributes to increased deaths owing to inadequate care.

#### Case in point: malaria

An information model to study and apply interventions in an ongoing epidemic such as malaria was discussed. Malaria is a unique disease that is confined to humans and mosquitoes; there is no animal model. Understanding the ecosystem, vector biology, immunoproteome of the parasite, and development of diagnostics has aided in the discernment of patterns of malaria in certain parts of Africa. This information has been utilized for malaria prediction and control.

#### Case in point: food systems

Using existing food safety surveillance systems in the United States as models, it may be possible to predict an impending food-borne disease outbreak. An efficient application of mathematical models based on several years of seasonal outbreaks and detailed data on the food-supply chain have made this possible. The United States has a robust and well-developed public health infrastructure for tracking food-borne disease outbreaks. There are many new diagnostic tools for characterizing disease pathogens. The team emphasized the need to look for social interactions and patterns of disease to predict outbreaks. Applications of novel technologies such as microbiome analysis to food-borne outbreaks were discussed as powerful tools in risk analysis and mitigation.

### The vision for safe food in a food system

This workshop was jointly convened by the iCOMOS, the Minnesota Governor's Food Safety and Defense Task Force, and the Minnesota Department of Agriculture, with participants from industry, academia, and regulatory bodies. To frame the discussions, the workshop began with three animal producers working at three very different scales: purely local swine production, regional poultry production with national distribution, and global poultry production and distribution. Each presenter described their own system and challenges. Given the dramatic differences in scale and complexity of the three systems, most striking was the similarity in the needs, challenges, and future risks that the three firms are trying to manage. Two overarching challenges, which drive many of the others, are limited public understanding of food systems, in some cases magnified by the reduced transparency of the food systems, and a public and legal expectation of zero risk. As a result, each of the firms faced issues around the implementation of animal welfare and food-safety systems and effective communication of their practices to the public and, in some cases, regulators. Given increasing concerns of antibiotic-resistant bacteria, the firms were also facing challenges in how to meet animal welfare and food-safety goals with reduced availability of antibiotics as a tool to maintain animal health.

Interestingly, the three producers working at different scales viewed their relationship to each other as a normal part of consumer choice and not as directly competitive. There were important differences between scales, with the small-scale producer always being challenged to secure consistent access to appropriate slaughter facilities, who favor large-size producers. Also, small scale limits market access to direct marketing of products, with less predictability. Unique at the regional level was the struggle to maintain input costs at a low level so that they remain competitive with health and health care. At the global scale, the producer had to deal with navigating global trade systems as well as conflicting regulatory requirements across countries.

Following the presentations, the audience divided into two sections, with one looking at food quality and the other discussing food sustainability, sufficiency, and security. Both groups had a combination of industry, regulatory, and academic participants and worked to gain consensus on the most important challenges facing the food system. The food-quality group identified food safety as the primary threat to food quality, with a focus on *Salmonella*. The group discussed several cases as illustrations of their key concerns, from the infection of food animals to cross-contamination during processing and at the point of consumption. There was discussion and the group outlined some of the reasons for food-safety threats, including contamination during transport, novel host–pathogen introduction pathways, risk assessments based on insufficient scientific evidence, and supply chain complexity. Effective risk communication was discussed as a path to overcome the limited transparency of the food system, which has led to unrealistic expectations of zero risk and poor dissemination of information about real food safety to the public. In addition, the participants saw that there is a disconnect between liability and responsibility that often comes up in cases of food-borne illness, a gap that impedes the appropriate improvements in overall food-safety risk management that could lead to fewer illnesses.

The food sustainability, sufficiency, and security discussion session developed a list of the expectations for food in the developed world and enumerated forces that affect those expectations. The group then discussed the sustainability of food and consumer attitudes about food production. Among the unique challenges to meeting consumer expectations of a sustainable, sufficient, and secure food system are the resistance to production agriculture systems in certain communities, the *not in my back yard* (NIMBY) syndrome, and food waste that contributes to an insufficient supply of food for some populations in spite of an excess of primary food production. Finally, the group focused on solutions to meet or alter consumer expectations of food sufficiency in a sustainable model including availability, affordability, dietary choice, and food waste.

Comparing notes from the two groups, it was instructive to see how similar the main points were, as well as the absence of certain anticipated elements. The fundamental challenges were the same for both groups and for all three scales of production. The differences in the specifics, drivers, and potential solutions were framed by the different topical charge to each group. While maintaining affordability for consumers was also discussed, the cost of food safety was not, other than the disconnect between liability and responsibility. The firms represented see food safety as a fundamental requirement for doing business. The challenge, whether for a single farm producer, a regional producer, or a global producer, is managing across the breadth of supply chains and the range of requirements, expectations, and knowledge of those that participate in and benefit from the food systems the firms operate.

### NIH-iCOMOS workshop on grantsmanship: strategies for success in a research career

This workshop provided a forum for information exchange in areas to assist both early and established investigators in the development of strategies to identify new funding opportunities; secure research funds; explore new training paradigms to meet future research needs; and identify institutional support for investigators. Hortencia Hornbeak, associate director for scientific review and policy at the NIAID, underscored the importance of identifying distinct niches in a field to target a project. The funding agency's goals should align well with the research topic and the research focus should align with the scientific mission of the funding institution. Communication is essential to successful applications, particularly between the applicant and the program officer of the funding agency. Review by colleagues will enhance the clarity, importance, and economy of the proposal. Collaborators greatly strengthen grants, and there is growing interest in the concept of program projects.

#### Journey from an idea to a successful application

According to Matthew J. Fenton, director of the Division of Extramural Activities at the NIAID, the NIH has historically promoted hypothesis-driven science, although it is slowly changing, with perhaps greater emphasis on innovative, high-impact, high-risk, high-reward proposals. Both technology-driven and descriptive studies are now welcomed by the NIH for consideration of funding. The specific aims of the proposal must be focused and complementary to each other, but not interdependent. It is essential for a successful submission that the grant is relevant, justified, progressive, and clearly demonstrates that the researcher has the expertise necessary to do the research. A detailed discussion was provided on preparing a competitive grant application by (1) having colleagues review the proposal before submission; (2) providing preliminary data to support each of the specific aims; and (3) demonstrating that the research team is integrative, complementary, and has the expertise to carry out the research. For reapplications, it is essential to address reviewer comments and assure that the grant is at least somewhat different and improved over the previous submission. R21 grants are meant to support high-risk, high-reward research and, as such, need much less preliminary data and are quite shorter in length. However, some NIH institutes are beginning to phase out R21 funding.

#### Establishing and maintaining collaborations panel: strategies for team science

The panel presented examples of successful collaborations. Peter Jackson highlighted the importance of communication and suggested strong integration of team members at all steps of the research process, including idea brainstorming and grant submission. He also promoted diversity and “prenups” regarding authorship, control of funding, and specimen ownership. Brooks Jackson highlighted the importance of choosing the right research (i.e., research that is impactful and advances the field). While he was a strong proponent of international collaborations, he recognized the challenges involved in global research projects and grant submissions. Bruce Blazar focused on how databases can greatly enhance research depth and promote collaboration, and recommended the following resources: the Clinical and Translational Science Institute (CTSI), MARCH, and biospecimen repositories. Each of the panelists underscored the importance of team science, whether as a local or international collaboration.

#### Training challenges panel

Not all PhD students will go on to get jobs in academia or industrial research, and it is not clear which institution is responsible for providing training to promote other options, according to Matthew Fenton. Fenton noted that the current trend in NIH support is for academic institutions via T32 and F30/31 grants; however, the NIH is expanding that approach to more interdisciplinary fields, as cutting-edge science is driven by the interaction of two or more disciplines. NIH-funded trainees are more successful than nontrainees at achieving subsequent funding from the NIH. The NIH budget is flat and it is predicted not to change in the foreseeable future. Ideas that integrate industry and academic nonresearch training needs, such as flexibility in research skills and teaching, are encouraged. Claudia Neuhauser noted that new skills that keep up with trends in big data are helpful. New and better analytical ways to train biomedical researchers to work with large data banks, starting at the undergraduate or high-school levels, are required. Better integration of biomedical and bioinformatics students is an excellent means to promote these training goals. Furthermore, she delineated the need to confront the fact that not all bioinformatics training should be a semester-long event. Short courses and workshops also are economical and effective means to connect people.

Personnel gaps were identified and focused on the physician–scientists that are an “endangered” species. Clifford Steer pointed out that the physician–scientist can provide important connections between researchers and patients. Similarly, professionals in other disciplines could provide linkages to science and should include not only training for MDs, but also DVMs, DDSs, and RNs. Multidisciplinary training and the grants that support it are essential to promoting one health initiatives. Furthermore, these grants must do a better job of increasing student diversity, stated Thomas Molitor. Still, meeting the challenges of training the next generation to address the complex challenges of the new millennium will be difficult. For example, Maura Donovan stated that private industries often seek skills that are different than in academia, and many traditional PhD students do not receive the training necessary for success in industry. She identified three areas of failure, including lack of knowledge of industry, no networking at the graduate level, and little training on how to successfully manage a career in industry. This hinders students in getting their first job in industry as well as in having a successful career. Students need access to a close link between academia and industry, which can be achieved by increasing the number of “expats” from industry in academia and creating forums where alumni can interact with current students.

During a question-and-discussion period, it was noted that the NIH has to integrate industry needs into their training grant programs. They also should consider allowing K awardees to have less than 75% research requirements to develop alternative means of salary support, but this is heavily dependent on which NIH institute is funding the K award. Furthermore, there must be increased flexibility in funding to promote scientists to get professional degrees or professional degree holders to get PhDs if there is to be an increase in physician–scientist numbers. Finally, to achieve the collaborative goal of One Medicine One Science, it is important to provide student-training opportunities that combine rigorous science with translational implementation in animal, human, or environmental health.

#### Gaining an administrative edge in a competitive world

James Casey, Jr., pre-award manager of the Office of Sponsored Programs at Carnegie Mellon University, and Pamela Webb, associate vice president of the Sponsored Projects Administration at UMN, ran this workshop. Organizational units that manage grant submission and administration can provide valuable assistance to research applicants. Keys to fully utilize these services include advance planning, inclusion of administrative staff early in the process, reviewing financial records monthly, providing feedback to show appreciation, and communicating with the administrative staff.

Researchers should be aware of any cost sharing needed in their research. There are three types to consider, including mandatory, voluntary committed, and no-strings cost sharing. Knowing the specific institutional policies on cost sharing and sub-awards or consultants can greatly increase the financial efficiency of research management. NIH budgets also need to be particularly well detailed and justified, as lack of justification makes budget reduction an easy target for the NIH budget review. For large project grants, it also may be economical to request support for an administrator.

#### New NIH initiatives and funding opportunities

Peter Jackson, branch chief of the AIDS Research Review Branch at the NIAID, explained that there are six major new NIH sources for funding: brain research through advancing innovative neurotechnologies (BRAIN), accelerated medicine, translating big data to knowledge, cancer immunotherapy, influenza prevention, and biomedical workforce improvement. Information on these programs can be found on the NIH website. Researchers are strongly encouraged to contact the program officers for information about specific requests for application (RFAs) and funding opportunity announcements (FOAs) for these projects. Also, the NIH is increasing small business paperwork relief (SBPR) and small business technology transfer (STTR) grant money availability by 2017. The NIH is also reevaluating indirect costs at the university level, and if they are reduced, more money will be available to support grants.

## Conclusions and lessons learned

The iCOMOS brought together a broad range of ideas and opinions to confront endemic and emerging threats to the health of animals, humans, and the environment. The ensuing presentations and discussions involved a diversity of knowledge disciplines and cultures. The common thread connecting people at every intersection was science with a common passion for solving problems.

As an example, shared curiosity and common interests enabled all attendees to grasp the significance of cultural change in farming practices on *Salmonella* evolution, resulting in new routes of human infection. Manure fertilization of food crops has been practiced for millennia. Recent innovations resulting in spray irrigation of vegetable crops in the United States, however, created a novel environment in which *Salmonella* evolved to grow on leaf surfaces. The adaptation spread throughout the *S. enterica* species so quickly that the trait went from unknown to ubiquitous in the late 1990s.

The act of engaging experts outside of one's area of expertise excites a mental ferment and reconsideration of previous opinions that can open new avenues of thought and action. If food poisoning events are due to plant-borne bacteria, then what are the implications for evolution and dissemination of antibiotic-resistance genes? Does feeding of antibiotics not used in humans to food animals at levels below minimal inhibitory concentrations result in drug-resistant pathogens in humans? Does increased animal production efficiency gained by nontherapeutic antibiotic use reduce agricultural demand for land and water, thus benefitting the environment? Does more efficient protein production increase affordability of nutritious food to humans? Science has a critical role to play in enlightening the discussion of these complex questions, in breaking down cultural and disciplinary silos, and in promoting public policy development based on knowledge.

The history of medical advancement is characterized largely by a linear scientific process leading from disease description, to pathogen or causation discovery, to a cure. The process has been wonderfully successful for stable host–pathogen models in closed systems, but it is challenged by complex, dynamic examples. Tuberculosis, malaria, and HIV exemplify host–pathogen relationships that are complex and demand significant scientific elucidation at multiple levels from the molecular to the community. The challenge to science and medicine is to find ways to characterize the human, animal, and environmental interactions so that change can be incorporated into predictive models.

Monkeypox is an interesting example of new disease whose threat to humans arose from eradication of an ecological competitor, smallpox virus. Smallpox virus dominated the human host niche of orthopoxviruses owing to highly efficient infection and transmission mechanisms, and by virtue of anti-smallpox virus immunity providing resistance to monkeypox virus. However, smallpox eradication and the ensuing loss of human immunity opened the door to monkeypox.^10^ Ecological modeling helped to reveal the apparent emergence of monkeypox and assist the medical and public health response. In a related vein of ecological complexity, thriving bee populations are required for pollination of two-thirds of the world's crop species. Commercial honey bee populations are in decline for various reasons, including emerging diseases and unintended consequences of pesticide use. Realistic ecological models incorporating transport movement and population mixing of commercial bee populations, combined with molecular genetics and genomics for selection of robust strains, can address existing honey bee declines while preparing for future challenges.

We live in a rapidly changing world characterized by global movement of humans, animals, trade, and food; by unparalleled environmental disruption and upheaval; and by irresolvable human conflict. The iCOMOS is a forum for melding the diverse elements of science and medicine that together can find common ground and solutions to these challenging and complex problems. In addition to sharing scientific knowledge and stimulating thought across diverse disciplines and cultures, the iCOMOS serves critical roles in networking and communication. It brings together scientists, medical experts, funding sponsors, and critical-need partners locally and globally for the purpose of bidirectional communication. What are the critical priorities in a vast ocean of needs? Where are the seeds of solutions in the universe of murky problems? Where is the engagement and advocacy without which informed public policies can hardly be developed?

Hence, involvement of funding sponsors, policy makers, and those who implement on the ground was an integral component of the iCOMOS. Here we are faced with the grand challenge of desire versus reality. Consumer choice, along with the ethics, values, and perceptions embedded within, is a powerful influence on public practice and policy. Thus, communication of competent science and medicine is essential. It is incumbent on scientists, physicians, veterinarians, and public health and policy makers to engage the public to provide objective knowledge. Knowledge is an important tool for improving the human condition, the animal condition, and the environmental condition. For this reason, the panel discussions and workshop elements of the iCOMOS played essential roles in assessing community needs for medical and scientific workforce development; for collaborative networking in defined areas of disease anticipation, food security, and one health strategies; and in assisting public policy decision making.

As this is being written, Ebola outbreaks have spread fear of the unknown around the world. Yet, Ebola and Marburg filoviruses have been known for decades. Why are there no countermeasures at this time? Where are the public health plans and policies? When might we become proactive instead of reactive? The iCOMOS meeting provides a forum to present and weigh the objective merits of competing health priorities and identify gaps in knowledge that threaten health security, so that major public policy implications can be discussed and decided with an underlying platform of facts. The meeting forum seeks to achieve this goal through integrative interactions across multiple platforms of knowledge and people, as shown earlier in Figure 1, and will form the basis of discussions for iCOMOS-2016.

## References

[1] United Nations Economic and Social Affairs Population Division (2014).

[2] Clifford DL (2013). Tuberculosis infection in wildlife from the Ruaha ecosystem Tanzania: implications for wildlife, domestic animals, and human health. Epidemiol. Infect.

[3] Roug A (2014). Comparison of intervention methods for reducing human exposure to *Mycobacterium bovis* through milk in pastoralist households of Tanzania. Prev. Vet. Med.

[4] Nguku PM (2010). An investigation of a major outbreak of Rift Valley fever in Kenya: 2006–2007. Am. J. Trop. Med. Hyg.

[5] Munyua P (2010). Rift Valley fever outbreak in livestock in Kenya, 2006–2007. Am. J. Trop. Med. Hyg.

[6] Anyamba A (2006). Developing global climate anomalies suggest potential disease risks for 2006–2007. Int J Health Geogr.

[7] Anyangu AS (2010). Risk factors for severe Rift Valley fever infection in Kenya, 2007. Am. J. Trop. Med. Hyg.

[8] Rich KM, Wanyoike F (2010). An assessment of the regional and national socio-economic impacts of the 2007 Rift Valley fever outbreak in Kenya. Am. J. Trop. Med. Hyg.

[9] Karesh WB (2012). Ecology of zoonoses: natural and unnatural histories. Lancet.

[10] Lloyd-Smith JO (2013). Vacated niches, competitive release and the community ecology of pathogen eradication. Philos. Trans. R Soc. Lond. B Biol. Sci.

[11] Blumberg S, Lloyd-Smith JO (2013). Inference of *R*_0_ and transmission heterogeneity from the size distribution of stuttering chains. PLoS Computational Biology.

[15] Lloyd-Smith JO (2009). Epidemic dynamics at the human-animal interface. Science.

[12] Palen J (2012). PEPFAR, health system strengthening, and promoting sustainability and country ownership. J. Acquir. Immune Defic. Syndr.

[13] Weston AD, Hood L (2004). Systems biology, proteomics, and the future of health care: toward predictive, preventative, and personalized medicine. J. Proteome Res.

[14] Sivapalasingam S (2001). Fresh produce: a growing cause of outbreaks of foodborne illness in the United States, 1973 through 1997. J Food Prot.

