# Supplementary material for: One Medicine One Science: a framework for exploring challenges at the intersection of animals, humans, and the environment
Source: Ann N Y Acad Sci. 2014 Dec 4;1334(1):26–44. doi: 10.1111/nyas.12601 (PMC4383647; doi:10.1111/nyas.12601)
Supplement: Supplementary file 1 [file nyas1334-0026-sd1.pdf]

Sunday, April 27

3:00 pm | Registration Opens
5:00 pm – 8:30 pm | **Opening Session**
5:00 pm – 5:05 pm | *Conference Welcome and Introductions*
Srirama Rao, *Associate Dean for Research, College of Veterinary Medicine, University of Minnesota*

5:05 pm – 5:15 pm | *University Welcome*
Eric Kaler, *President, University of Minnesota*

Brian Herman, *Vice President for Research, University of Minnesota*

5:15 pm – 5:30 pm | *A Unified Vision of One Medicine One Science: The Science Behind One Health*
Trevor Ames, *Dean, College of Veterinary Medicine, University of Minnesota*

5:30 pm-7:00 pm | *Global Challenges at the Interface of Animals, Humans and the Environment:*
Role of Science and Discovery in the Pursuit of One Health
Panel Vignettes and Moderated Discussion
Moderator:
Maggie Koerth-Baker, *Columnist, The New York Times Magazine; Science Editor, BoingBoing*

Panelists:

Peter Agre, *Nobel Laureate, University Professor & Director, Johns Hopkins Malaria Research Institute, Johns Hopkins University*

William Bazeyo, *Dean, School of Public Health, Makerere University, Kampala, Uganda*

David M. Morens, *Senior Advisor to the Director, National Institute of Allergy and Infectious Diseases, (NIAID), National Institutes of Health (NIH)*

Lertrak Srikitjakarn, *Professor and Dean Emeritus, Faculty of Veterinary Medicine, Chiang Mai University*

Samuel Thevasagayam, *Deputy Director, Agriculture Development, Livestock, Bill & Melinda Gates Foundation*

7:00 pm – 8:30 pm | Opening Reception

Monday, April 28 Role of Science in Solving Emerging Health Threats at the Interface

7:00 am – 8:00 am	Continental Breakfast
8:00 am – 12:00 pm	**The Science of Disease Recognition at the Human-Animal-Environment Interface**
8:00 am – 8:15 am	Welcome and Introduction<br>**Brooks Jackson**, *Medical School Dean and Vice President for Health Sciences, University of Minnesota*
8:15 am – 9:00 am	*Aquaporin Water Channels: From Atomic Structure to Malaria*<br>**Peter Agre**, *Nobel Laureate, University Professor & Director, Johns Hopkins Malaria Research Institute, Johns Hopkins University*
9:00 am – 9:05 am	*Role of Science in One Health*<br>Session Chairs:<br>**Beth Virnig**, *Associate Dean for Research and Professor, School of Public Health, University of Minnesota*<br>**Bill Stauffer**, *Associate Professor of Infectious Disease, Medical School, University of Minnesota*
9:05 am – 9:35 am	*Recognition of Disease Emergence at the Interface: Influenza*<br>**Richard Webby**, *Director, St. Jude Children's Research Hospital, Memphis, TN*
9:35 am – 10:05 am	*Postcards from the Destabilized Edge: Novel Pathogen Emergence*<br>**M. Kariuki Njenga**, *Head, Integrated Human-Animal Health (One Health) Program, United States' Centers for Disease Control and Prevention (CDC)-Kenya, Nairobi, Kenya*
10:05 am – 10:20 am	Break
10:20 am – 10:50 am	*The Ecology of Tuberculosis in Pastoralist Communities*<br>**Rudovick Reuben Kazwala**, *Professor, Department of Veterinary Medicine and Public Health, Sokoine University of Agriculture, Tanzania*
10:50 am – 11:10 am	*Evidence Based Approach to One Health: A Perspective from the Bill and Melinda Gates Foundation*<br>**Samuel Thevasagayam**, *Deputy Director, Agricultural Development, Livestock, Bill & Melinda Gates Foundation*
11:10 am – 11:30 am	*NIAID Efforts in Infectious Diseases at the Animal-Human Interface*<br>**Tina Parker**, *Program Officer, Division of Microbiology and Infectious Disease (DMID), National Institute of Allergy and Infectious Diseases (NIAID),National Institutes of Health (NIH)*
11:30 am – 12:00 pm	**Panel Q & A** (Webby, Njenga, Kazwala, Thevasagayam, Parker)
12:00 pm – 1:00 pm	Lunch

Monday, April 28

1:00 pm – 5:15 pm	**Evolving Solutions to the Changing Infectious Disease Horizon**
1:00 pm – 1:05 pm	Session Chair: **Clifford J. Steer**, *Professor, Gastroenterology, Hepatology and Nutrition, Medical School, University of Minnesota*
1:05 pm – 1:35 pm	*Vaccine Prevention Strategies in a Changing World*<br>**Mark Feinberg**, *Vice President and Chief Public Health Officer, Merck Vaccines, Merck & Co., Inc.*
1:35 pm – 2:05 pm	*Strategies for AIDS Interventions in Africa*<br>**Phyllis Kanki**, *Professor of Immunology and Infectious Diseases, Department of Immunology and Infectious Disease, Harvard School of Public Health*
2:05 pm – 2:35 pm	*Monkeypox Emergence in the Aftermath of Small Pox Eradication: Ecological Opportunity & Evolutionary Potential*<br>**James Lloyd-Smith**, *Associate Professor, Department of Ecology and Evolutionary Biology, University of California, Los Angeles*
2:35 pm – 2:55 pm	*Social, Cultural, and Political Solutions to Emerging Disease-Video Presentation*<br>**David Nabarro**, *Special Representative for Food Security and Nutrition, United Nations*
2:55 pm – 3:25 pm	**Panel Q & A** (Feinberg, Kanki, Lloyd-Smith)
3:25 pm – 3:35 pm	Break
3:35 pm – 5:15 pm	*Scientific Partnerships for Solving the Global Health Crisis – Moderated Panel Discussion*<br>Moderator: **Brian Herman**, *Vice President for Research, University of Minnesota*<br><br>Panelists:<br>**Andrew Clements**, *Deputy Director of Pandemic Influenza and Other Emerging Threats Program, US Agency for International Development (USAID)*<br>**Carole Heilman**, *Director, Division of Microbiology and Infectious Disease (DMID), National Institute of Allergy and Infectious Diseases (NIAID), National Institutes of Health*<br>**Sonny Ramaswamy**, *Director, National Institute of Food and Agriculture (NIFA), US Department of Agriculture*<br>**Samuel Thevasagayam**, *Deputy Director, Agricultural Development, Livestock, Bill & Melinda Gates Foundation*
6:00 pm – 8:15 pm	Evening at the McNamara Alumni Center (across street from The Commons Hotel)
6:00 pm – 6:30 pm	Social hour
6:30 pm – 7:30 pm	Dinner
7:30 pm – 8:15 pm	*Food Animal Domestication and Human Advancement*<br>Introduction: **Karen Hanson**, *Senior Vice President for Academic Affairs and Provost, University of Minnesota*<br>Speaker: **Sonny Ramaswamy**, *Director, National Institute of Food and Agriculture (NIFA), US Department of Agriculture*

Tuesday, April 29 Alignment of Safe Food, Sustainable Production, and Consumer Attitudes

7:00 am – 8:00 am	Continental Breakfast
8:00 am – 12:00 pm	**The Grand Challenges of Food Production**
8:00 am – 8:05 am	Introduction: **Allen Levine**, *Professor, Department of Food Science and Nutrition, College of Food, Agricultural and Natural Resource Sciences, University of Minnesota*
8:05 am – 8:30 am	*PepsiCo Global R&D: Addressing Today's and Tomorrow's Food and Beverage Challenges and Opportunities*<br>**Mehmood Khan**, *Executive Vice President and Chief Scientific Officer, PepsiCo*
8:30 am – 10:00 am	*Meeting the Conflicting Challenges of Feeding the World – Moderated Panel Discussion*<br>Moderators:<br>**Allen Levine**, *Professor, Department of Food Science and Nutrition, College of Food, Agricultural and Natural Resource Sciences, University of Minnesota*<br>**Shaun Kennedy**, *Associate Professor of Food Systems, Department of Veterinary Population Medicine, College of Veterinary Medicine, University of Minnesota*<br>Panelists:<br>**Sarah Brew**, *Partner, Food Litigation and Regulatory Practice Lead, Faegre Baker Daniels*<br>**Erin Fitzgerald**, *Senior Vice President, Sustainability of Dairy Innovation Center*<br>**Mehmood Khan**, *Executive Vice President and Chief Scientific Officer, Global Research and Development, PepsiCo*<br>**Mike Robach**, *Vice President, Corporate Food Safety, Quality & Regulatory Affairs, Cargill, Inc.*
10:00 am – 10:15 am	Break<br>Session Chair: **John Finnegan**, *Dean, School of Public Health, University of Minnesota*
10:15 am – 10:45 am	*The Reality of Food Production Needs Globally*<br>**Jonathan Foley**, *Director, Institute on the Environment (IonE), Professor and McKnight Presidential Chair of Global Environment and Sustainability, University of Minnesota*
10:45 am – 11:15 am	*The Law of Unintended Consequences: Bee Health Affects Human Health*<br>**Marla Spivak**, *Distinguished McKnight Professor, Department of Entomology, College of Food, Agricultural and Natural Resource Sciences, University of Minnesota*
11:15 am – 11:45 pm	*Linking Antibiotic Resistance in Animals and Humans*<br>**Henrik Wegener**, *Provost, Chief Academic Officer, Vice Executive President, Technical University of Denmark*
11:45 am – 12:00 pm	**Panel Q & A** (Foley, Spivak, Wegener)

Tuesday, April 29

12:00 pm – 1:30 pm	Lunch – *The Anthropology of Food: Consequences for Humans, Animals and the Environment*<br>Introduction:<br>**Gary Reineccius,** *Department Head, Food Science and Nutrition, College of Food, Agricultural and Natural Resource Sciences, University of Minnesota*<br>**Andrew Zimmern**, *James Beard Award-winning TV personality, chef, food writer, and teacher*
1:30 pm – 4:35 pm	**The Grand Challenges in Food Safety**
1:30 pm – 1:35 pm	Session Chair: **Will Hueston**, *Professor, College of Veterinary Medicine, University of Minnesota*
1:35 pm – 2:05 pm	*The Wicked Problem of Food Safety*<br>**Catherine Woteki**, *USDA Chief Scientist and Under Secretary for Research, Education and Economics*
2:05 pm – 2:35 pm	*Microbial Evolution Leading to Food-Borne Illness*<br>**Stanley Maloy**, *Dean, College of Sciences, and Professor of Microbiology, San Diego State University*
2:35 pm – 2:50 pm	Break
2:50 pm – 3:20 pm	*How Safe Do We Really Want to Be? The Paradox*<br>**Michael Apley**, *Professor and Section Head, Production Medicine/Clinical Pharmacology, College of Veterinary Medicine, Kansas State University*
3:20 pm – 3:50 pm	*Consumer Attitudes Toward Emerging Technologies and Food*<br>**Jennifer Kuzma**, *Goodnight–NC GSK Foundation Distinguished Professor in Social Sciences and Co-Director, Genetic Engineering and Socciety Center, North Carolina State University*
3:50 am – 4:30 pm	**Panel Q & A** (Woteki, Maloy, Apley, Kuzma)
4:30 pm – 4:35 pm	*Perspective of the Future*<br>**Michael Murtaugh**, *Professor of Molecular Biology, College of Veterinary Medicine, University of Minnesota*

Wednesday, April 30

Grantsmanship in a Changing Environment Workshop

MODERATORS & PRESENTERS:

**Dr. Bruce Blazar**	*Regents Professor, Medical School, Associate Vice President, AHC Clinical Research, University of Minnesota*
**James J Casey, Jr.**	*J.D., Pre-Award Manager, Office of Sponsored Programs, Carnegie Mellon University*
**Dr. Maura Donovan**	*Executive Director, Office of University Economic Development, University of Minnesota*
**Dr. Matthew Fenton**	*Director Division of Extramural Activities, National Institute of Allergy & Infectious Diseases, (NIAID), National Institutes of Health (NIH)*
**Dr. Hortencia Hornbeak**	*Associate Director for Scientific Review and Policy, National Institute of Allergy & Infectious Diseases, (NIAID), National Institutes of Health (NIH)*
**Dr. Brooks Jackson**	*Dean of Medical School, & VP for Health Sciences, University of Minnesota*
**Dr. Peter Jackson**	*Branch Chief, AIDS Research Review Branch, National Institute of Allergy & Infectious Diseases, (NIAID), National Institutes of Health (NIH)*
**Dr. Thomas Molitor**	*Distinguished Teaching Professor & Chair, Veterinary Population Medicine, University of Minnesota*
**Dr. Claudia Neuhauser**	*Director, University of Minnesota Informatics Institute*
**Dr. Clifford Steer**	*Professor, Gastroenterology, Hepatology & Nutrition, Medical School, University of Minnesota*
**Pamela Webb**	*Associate Vice President, Sponsored Projects Administration, University of Minnesota*

DESCRIPTION: The workshop led by NIH grants program directors and administrators and leading researchers in areas of infectious disease and one health will provide a forum for information exchange in the following areas: assist early and established investigators in the development of strategies that would enable them to secure research funds; strategies for submitting a successful application; identify new funding opportunities; SBIR/STTR; highlight international collaborative /partner opportunities; explore new training paradigms to meet future research needs; and Institutional support (e.g. in identifying funding opportunities for investigators). This interactive workshop will provide many opportunities for participants to ask questions and seek additional information from the experts.

AUDIENCE: Scientists, clinicians, public and animal health researchers, grant administrators.

OUTCOME: Participants will leave the workshop with knowledge, materials and contact information useful for immediate implementation in organization of research teams and grant submission. (CDs containing resumes of speakers, and all presentations will be provided so that participants can review and reinforce what was discussed at the Workshop. Other resources that will be provided include: funding opportunities websites, references on strategies for collaboration, and research administrative sites).

SCIENTIFIC VALUE: Direct interactions and input from NIAID and University grants program administrators, faculty and staff with expertise in various fields including infectious disease and one health.

LOCATION: The Commons Hotel

AGENDA

7:00 am - 8:00 am	Registration & Breakfast
8:00 am- 8:05 am	Welcome-Srirama Rao
8:00 am - 8:45 am	Strategies for Success - Hortencia Hornbeak
8:45 am - 9:30 am	Journey from an Idea to a Successful Application-Matthew Fenton
9:30 am - 10:30 am	Establishing & Maintaining Collaborations – Strategies for "Team Science" – Peter Jackson; Brooks Jackson and Bruce Blazar
10:30 am - 10:45 am	Break
10:45 am - 12:00 pm	Training Challenges (panel discussion) - Matthew Fenton, Claudia Neuhauer, Cliff Steer, Tom Molitor and Maura Donovan
12:00 pm - 1:15 pm	Lunch
1:15 pm - 1:45 pm	New NIH Initiatives & Funding Opportunities – Peter Jackson
1:45 pm - 2:45 pm	Gaining an Administrative Edge in a Competitive World – Pamela Webb & James J. Casey Jr.
2:45 pm - 4:00 pm	Mock Peer Review – Peter Jackson, James J. Casey Jr. , Matthew Fenton, Cliff Steer, Hortencia Hornbeak
4:00 pm	Adjourn
4:00 pm - 5:30 pm	Opportunity to meet with speakers individually or in a small group (2-4) Speakers: Fenton; Hornbeak; Jackson; Casey and Webb. **These meetings will require appointments;** sign-up sheets will be available (15 mins per person/group)

Wednesday, April 30

Is it Possible to Predict the Next Outbreak Threat? Workshop

CONVENERS:

Srinand Sreevatsan, *Professor, Veterinary Population Medicine, College of Veterinary Medicine, University of Minnesota*

Chandy John, *Director, Division of Global Pediatrics; Professor of Pediatrics and Medicine, Medical School, University of Minnesota*

David Boulware, *Associate Professor & Associate Director of Global Health Programs in Internal Medicine, Medical School, University of Minnesota*

Matteo Convertino, *Assistant Professor, Division of Environmental Health Sciences, School of Public Health, University of Minnesota*

Dan Knights, *Assistant Professor, Department of Computer Science & Engineering and the Biotechnology Institute, University of Minnesota*

Michael Osterholm, *Director, Center for Infectious Disease Research & Policy, School of Public Health, University of Minnesota*

DESCRIPTION: This workshop will challenge the existing paradigm (tools, technologies, systems used) in infectious disease emergence and/or reemergence based on an analytical review of key concepts. Participants will engage with mathematical modelers, field epidemiologists and bench scientists in interactive and formative sessions to discuss challenging situations and problems in the thematic areas of (1) mathematical models of patterns of infectious disease as a predictor of new events, (2) modeling pathogen evolution to predict pandemics – are the existing approaches sufficient?, (3) technological advances – What is the resolution needed for discovery of emergent events? (4) the computational challenge - biocomputation as an aid in pathogen discovery, and (5) case-in-point - synthesis of the challenge of predict.

AUDIENCE: Front-line surveillance and public health personnel, epidemiologists, veterinarians, disease specialists, regulatory scientists.

OUTCOME: An analytical review paper on the future of infectious disease surveillance and monitoring. New ideas to be assembled into a program project.

TIMELINE: Completion of white paper within 3 months of the workshop.

SCIENTIFIC VALUE:
Demonstration of the importance of scientifically valid data in development of evidence-based policies for disease control.

LOCATION: The Commons Hotel

AGENDA

8:30 am - 9:00 am Registration & Continental Breakfast
9:00 am – 9:30 am Welcome and Introductions
9:30 am - 11:30 am Interactive Discussions

– Thematic leads will give a 10-15 minute introduction and the contribution of their discipline to the improvement of predicting pathogen emergence. This will be followed by an interactive discussion.
- Chandy John, David Boulware, Matteo Convertino, Dan Knights, Srinand Sreevatsan

1. Optimal Multiscale Prediction, Detection and Response: Complexity Science as Cyber-infrastructure for Population Health (Matteo Convertino)
2. Modeling Pathogen Evolution to Predict Pandemics. Are the existing approaches sufficient to capture re-emergence? (Srinand Sreevatsan)
3. Technological Advances. What is the resolution needed for discovery of emergent events? How does mathematical modeling tie-in with technological advances to aid in detection of pathogen emergence? (Srinand Sreevatsan)
4. Computational Challenge: Biocomputation as an Aid in Pathogen Discovery (Dan Knights)

11:30 am - 12:15 pm Case-in-Point: Synthesis of the Challenge of New Pathogen Incursion

1. Can we use biomarkers of host immunity to predict risk of infectious disease? (Chandy John)
2. Gain in Function: Prediction of Pandemics (Michael Osterholm)
3. Challenges in Food Safety in Predicting Outbreaks (David Boulware)

12:15 pm - 12:30 pm Wrap-up
12:30 pm - 2:30 pm Lunch and Networking

Wednesday, April 30

Vision for Safe Food Systems From Local to Global Workshop

CONVENERS:

Heidi Kassenborg, *Director, Dairy Food Inspection Division, MN Department of Agriculture and President of the Association of Food and Drug Officials*

Dave Read, *Assistant Director, Dairy Food Inspection Division, MN Department of Agriculture and President of the Association for Food and Drug Officials*

Joe Scimeca, *Director of Food Regulatory Affairs, Cargill Inc. and Chair, Minnesota Food Safety and Defense Task Force*

Shaun Kennedy, *Associate Professor of Food Systems, Veterinary Population Medicine, University of Minnesota*

DESCRIPTION: This workshop is a collaborative program with the Minnesota Food Safety and Defense Task Force, The Minnesota Department of Agriculture and the University of Minnesota.

Consumers get products from a wide range of food systems that operate on local, regional and global scales. The food industry imports from local, regional and global producers and exports to distributors acting on all scales. Each scale has its own advantages and challenges when it comes to food safety. Importantly, each scale can benefit from innovations in the other systems. Presentations will consider the current state of food safety, predicted demands on food systems, resources needed to maintain food quality in the future, innovations in the pipeline, and the impact of consumer expectations on food security. Emphasis will be on meat and eggs at local, upper Midwest region, national and global levels. Interactive discussions will involve participants on how future food systems might look and the resulting opportunities, risks and benefits across the scales.

AUDIENCE: Food producers, processors, distributors and retailers; veterinary and public health regulators; academics; legislators and NGO's interested in food systems.

OUTCOME: A white paper on Food Safety vision across scales that 1) identifies near-term and long-term problems for food production, food quality and food safety; 2) provide insights into current promising research and research directions, and 3) creates a vision of innovation, policy and implementation needs.

TIMELINE: The workshop brings expertise and perspective from iCOMOS speakers and participants into an existing partnership (Governor's taskforce on food safety) that is expected to continue after the session.

SCIENTIFIC VALUE: Bringing to focus a strategy for evidence based policy and regulation for food production, quality and safety.

LOCATION: Freeman Building, 635 Robert St. N., St. Paul, Minnesota – Transportation provided round-trip to/from The Commons Hotel

AGENDA

8:00 am - 8:30 am	Registration & Continental Breakfast
8:30 am - 9:00 am	Travel to Freeman Building
9:00 am - 9:30 am	Welcome and Introductions<br>**Joe Scimeca**, Director of Food Regulatory Affairs, Cargill Inc.<br>**Heidi Kassenborg**, Director, Dairy & Food Inspection Division, Minnesota Department of Agriculture
9:30 am - 10:30 am	Local/Regional/Global Systems Examined<br>Local-**Karen Weiss,** Little Foot Farm<br>Regional-<br>Global-**Todd McAloon**, Vice President, Global Poultry Food Safety and Quality, Cargill Meat Solutions
10:30 am - 11:30 am	Panel Q & A
11:30 am - 12:15 pm	Breakout Discussions:<br>Framing the Challenges of Food Safety & Quality; Security & Sufficiency; Consumer Expectations
12:15 pm - 12:45 pm	Breakout Group Reports
12:45 pm - 1: 15 pm	Lunch
1:15 pm - 2:30 pm	Breakout Discussions: Exploring Solutions
2:30 pm - 3:15 pm	Breakout Group Reports
3:15 pm - 3:30 pm	Wrap-up
3:30 pm - 4:00 pm	Travel to The Commons

Wednesday, April 30

One Health Partnerships: The Reality on the Ground Workshop

CONVENERS:

Beth Virnig, *Associate Dean for Research and Professor, School of Public Health, University of Minnesota*

Will Hueston, *Professor, College of Veterinary Medicine, University of Minnesota*

DESCRIPTION: One Health in the 'real world' takes people, time, commitment and collaborations. We are excited to offer a one day workshop that will explore One Health from the concept through the everyday realities on the ground.

We will commence with a plenary session addressing what One Health is and the actors who make the One Health system work. Following the morning plenary session we will hit the ground for an on-site panel discussion at the Minnesota State Fairgrounds to address animal human interactions in public settings.

We will end the afternoon in an interactive learning room where we will delve into the realities of how One Health works in the 'real world' and how we can build on this with synthesis of insights and improving One Health partnerships. Workshop participants will have the opportunity to carry these insights forward in a published synthesis document that can be used to inform stakeholders who bring One Health to the realities of disease detection, cure, and prevention.

AUDIENCE: Clinicians; public and animal health officials; and academicians

OUTCOME: Generation of a guideline document on One health partnership best practices

LOCATION: Animal Science/Veterinary Medicine Building, 1988 Fitch Ave, St. Paul Campus, University of Minnesota-Transportation provided round-trip to and from The Commons Hotel

	SESSION TITLE	LOCATION	ACTIVITY
7:00 am-7:30 am	Travel to Workshop Location	The Commons Hotel	- Bused from Hotel to AS/VM Building
7:30 am-8:00 am	Registration and Continental Breakfast		
8:00 am-10:00 am	One Health approaches & the actors that make it work	AS/VM Active Learning Classroom	- Introductions & icebreaker activity<br>- Review logistics and timeline<br>- Set the stage<br>- Interactive panel discussion
10:00 am-10:15 am	Health Break	Foyer	- Coffee / tea, snack
10:15 am-10:45 am	Introduction to human, animaland environmental interactions in public settings	AS/VM Active Learning Classroom	- Example disease and risks
10:45 am-11:30 am	Information Collection and Speed Networking	AS/VM Active Learning Classroom	- Intro to speed networking<br>- Speed networking activity<br>- Set-up for after lunch state fair sites
11:30 am-12:15 pm	Lunch	Pomeroy 215	- Sustenance, socializing & networking
12:15 pm-12:45 pm	Travel to State Fair		- Bused from AS/VM to State Fair
12:45 pm-2:45 pm	Interactive case studies and MN State Fair field assignments	Minnesota State Fairgrounds	- Visit field sites
2:45 pm-3:15 pm	Health Break and Travel to AS/VM		- Bused from State Fair to AS/VM
3:15 pm-3:55 pm	Breakout sessions: Synthesis of insights to improve One Health partnerships	AS/VM Active Learning Classroom	- Group Work
3:55 pm-4:10 pm	Group Reports	AS/VM Active Learning Classroom	- Groups report out
4:10 pm-4:40 pm	National and state guidelines for human and animal interactions in public settings		- National guidelines for PH interactions discussion
4:40 pm-5:00 pm	Final synthesis and evaluation	AS/VM Active Learning Classroom	- Synthesis, feedback and evaluation
5:00 pm-5:30 pm	Travel to The Commons		- Bused from AS/VM Building to Hotel
